# Placental Growth Factor (PlGF) Between 12 + 0–20 + 6 Weeks' Gestation and Perinatal Outcomes in a High Prevalent Diabetes Population: A Retrospective Cohort Study

**DOI:** 10.1111/1471-0528.70076

**Published:** 2025-11-05

**Authors:** Genevieve M. Dietrich, Adrielle P. Souza Lira, Eman Ramadan, Adewumi Adanlawo, Ernesto A. Figueiro‐Filho

**Affiliations:** ^1^ College of Medicine University of Saskatchewan Regina Saskatchewan Canada; ^2^ Community Health and Epidemiology – College of Medicine University of Saskatchewan Regina Saskatchewan Canada; ^3^ Fetal Assessment Unit, Regina General Hospital – College of Medicine University of Saskatchewan Regina Saskatchewan Canada

Pre‐eclampsia and placental dysfunction are characterised by an imbalance of angiogenic factors: maternal placental growth factor (PlGF) concentrations decline early and the antiangiogenic soluble fms‐like tyrosine kinase1 (sFlt1) rises later [1, 2]. In a case–control study, women who subsequently developed pre‐eclampsia had significantly lower PlGF levels from as early as 13–16 weeks of gestation (mean 90 pg/mL vs. 142 pg/mL in controls, *p* = 0.01) [2]. Increased levels of sFlt1 preceded clinical disease by approximately 5 weeks [2]. Therefore, depressed PlGF occurs first, followed by a subsequent sFlt1 rise. Relying solely on the sFlt1/PlGF ratio may miss early placental dysfunction [2]. Large prospective cohorts have shown that PlGF measured at 14–16 and 18–20 weeks is about half that of controls in pregnancies destined to deliver < 34 weeks with pre‐eclampsia or foetal growth restriction [1]. A recent review emphasised that PlGF and sFlt1 are ‘powerful new tools to guide obstetric and medical care’ [3]. Our group recently implemented PlGF testing across 12–36 weeks in a tertiary Western Canadian centre and reported high negative predictive values (> 90%) for pre‐eclampsia and preterm birth [4]. However, there remains limited evidence focusing specifically on the early second trimester in high‐risk populations such as those with a high prevalence of diabetes. We therefore aimed to evaluate maternal PlGF at 12 + 0–20 + 6 weeks' gestation in relation to pre‐eclampsia, preterm birth and low birthweight, using gestational age adjusted centiles rather than fixed thresholds.

This retrospective cohort study was conducted at the Fetal Assessment Unit of the Regina General Hospital, Regina, Saskatchewan, Canada. Institutional ethics approval was obtained (REB #Bio3702). The cohort comprised all pregnant individuals who underwent PlGF testing between December 2021 and September 2025 at 12 + 0–20 + 6 weeks' gestation and subsequently delivered during the study period. PlGF measurements were converted to gestational age‐specific centiles using published reference ranges [5]. Low PlGF was defined as < 10th centile and normal as ≥ 10th centile. Maternal characteristics (age, BMI, diabetes status), indications for testing, and outcomes were extracted from electronic records. BMI was categorised using WHO cutoffs: normal weight 18.5–24.9 kg/m^2^; overweight 25.0–29.9; obese I 30.0–34.9; obese II 35.0–39.9; obese III ≥ 40. Diabetes status (no diabetes, gestational diabetes [GDM], Type 1 DM or Type 2 DM) was recorded at the patient's first visit.

Outcomes were: (1) pre‐eclampsia (diagnosis recorded in the medical record), (2) preterm birth (PTB) < 37 weeks, and (3) low birthweight < 2500 g. Birthweight was also analysed as a continuous variable. Odds ratios (OR) with 95% confidence intervals (CI) were computed comparing low versus normal PlGF using Fisher's exact test for binary outcomes and Student's *t*‐test for continuous variables. Diagnostic metrics—sensitivity (S), specificity (E), positive predictive value (PPV), negative predictive value (NPV) and accuracy—were calculated for each outcome. Missing outcome data were treated as negative events. Analyses were performed using GraphPad Prism (version 10.6).

## 1 Study Population

**FIGURE 1:**
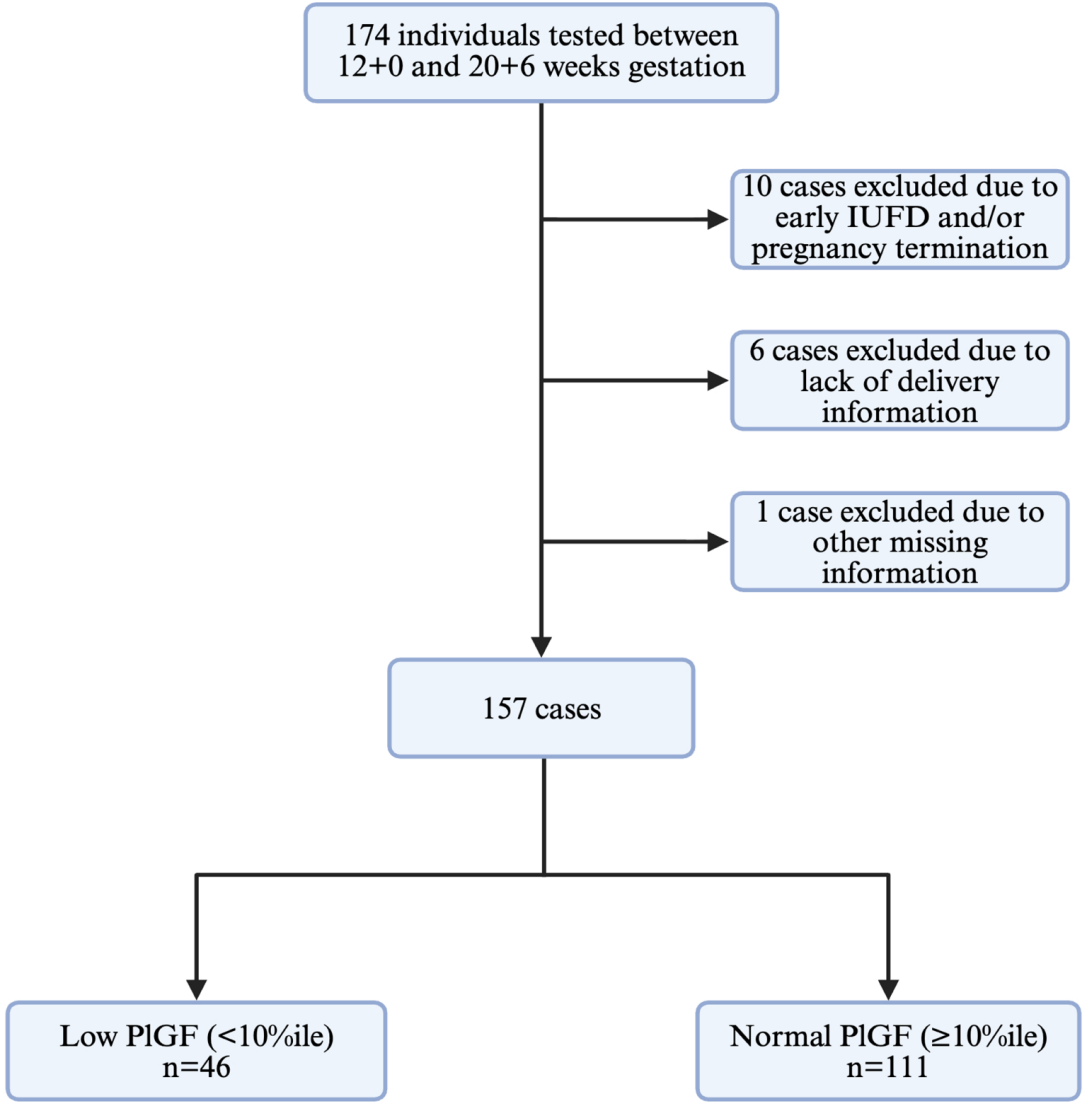
Flow diagram of population selection. A total of 174 individuals had PlGF tested between 12 + 0 and 20 + 6 weeks of gestation (17 cases were excluded from final analysis).

Among 174 individuals tested at 12 + 0–20 + 6 weeks during the study window, 10 cases were excluded due to early fetal demise or pregnancy termination, six cases were excluded due to a lack of delivery information, and one case was excluded due to other missing data (Figure 1). This gave 157 participants for analysis: 46 with low PlGF (< 10th percentile) and 111 with normal PlGF (≥ 10th percentile) (Figure 1).

The mean gestational age at testing was 17.2 ± 2.5 weeks. Maternal age did not differ between groups (31.8 ± 5.2 vs. 32.1 ± 5.7 years, *p* = 0.78). BMI was available for 76/157 and categorisation based on WHO guidelines is given in Table 1. Diabetes status was recorded for all cases. Low PlGF group comprised 24 patients without diabetes, 6 with GDM, 14 with Type 2 DM, 2 with Type 1 DM. Normal PlGF group included 53 patients without diabetes, 29 with GDM, 25 with Type 2 DM, and 4 with Type 1 DM.

**TABLE 1:** Maternal and perinatal outcomes by PlGF category (12 + 0–20 + 6 weeks' gestation).

Variable	Low PlGF (< 10th centile) (*n* = 46)	Normal PlGF (≥ 10th centile) (*n* = 111)	OR (95% CI)	*p*	Statistical test
Demographics					
Maternal age (years)	31.8 ± 5.2	32.1 ± 5.7	—	0.78	Student's *t*‐test
BMI			0.27	0.12	Fisher's exact
Normal weight	2/24 (8.3%)	13/52 (25.0%)	—	—	—
Overweight	4/24 (16.7%)	12/52 (23.1%)	—	—	—
Obese Class I	11/24 (45.8%)	15/52 (28.8%)	—	—	—
Obese Classes II–III	7/24 (29.2%)	12/52 (23.1%)	—	—	—
Diabetes			1.19	0.73	Fisher's exact
Non‐diabetic	24/46 (52.2%)	53/111 (47.7%)	—	—	—
GDM	6/46 (13.0%)	29/111 (26.1%)	—	—	—
Type 1 DM	2/46 (4.3%)	4/111 (3.6%)	—	—	—
Type 2 DM	14/46 (30.4%)	25/111 (22.5%)	—	—	—
Pre‐eclampsia	14/46 (30.4%)	15/111 (13.5%)	2.80 (1.21–6.38)	0.02	Fisher's exact
Preterm birth < 37 weeks	23/46 (50.0%)	24/111 (21.6%)	3.63 (1.69–7.44)	< 0.01	Fisher's exact
Low birthweight < 2500 g	9/41 (22.0%)	10/87 (11.5%)	2.17 (0.84–5.42)	0.18	Fisher's exact
Birthweight (g)	2977.4 ± 743.1 (*n* = 41)	3200.8 ± 690.3 (*n* = 87)	—	0.10	Student's *t*‐test

## 2 Outcomes

Table 1 summarises outcomes by PlGF category. Pre‐eclampsia developed in 14/46 (30.4%) low PlGF pregnancies versus 15/111 (13.5%) normal PlGF pregnancies (OR 2.80, 95% CI 1.21–6.38; *p* = 0.02). Preterm birth < 37 weeks occurred in 23/46 (50.0%) low PlGF pregnancies compared with 24/111 (21.6%) in the normal group (OR 3.63, 95% CI 1.69–7.44; *p* = < 0.01). Low birthweight < 2500 g was recorded in 9/41 (22.0%) low PlGF pregnancies versus 10/87 (11.5%) in the normal group (OR 2.17, 95% CI 0.84–5.42; *p* = 0.18). Mean birthweight was 2977.4 ± 743.1 g in the low PlGF group and 3200.8 ± 690.3 g in the normal PlGF group (*p* = 0.10).

## 3 Diagnostic Metrics

Table 2 summarises diagnostic performance. Normal PlGF had a negative predictive value of 86.5% for pre‐eclampsia, 88.5% for birthweight < 2500 g and 78.4% for PTB < 37 weeks.

**TABLE 2:** Sensitivity (S), specificity (E), positive predictive value (PPV), negative predictive value (NPV) and accuracy of low PlGF at 12 + 0–20 + 6 weeks.

Outcome	Low PlGF cases (yes/no)	Normal PlGF cases (yes/no)	S (%)	E (%)	PPV (%)	NPV (%)	Accuracy (%)
Pre‐eclampsia	14/32	15/96	48.3	75.0	30.4	86.5	70.1
Preterm birth < 37 weeks	23/23	24/87	48.9	79.1	50.0	78.4	70.1
Birthweight < 2500 g	9/32	10/77	47.4	70.6	22.0	88.5	67.2

This study confirms that low PlGF measured at 12 + 0–20 + 6 weeks' gestation is associated with pre‐eclampsia and preterm birth. Pre‐eclampsia occurred in 30% of low PlGF pregnancies versus 14% in the normal group (OR 2.80). NPV of normal PlGF for pre‐eclampsia was 86.5%. Preterm birth < 37 weeks occurred in 50% of low PlGF pregnancies versus 22% in the normal group (OR 3.63). NPV of normal PlGF for preterm birth was 78.4%. These findings align with mechanistic studies showing early decline in PlGF [2] and with evidence from large prospective cohorts where median PlGF at 14–20 weeks was half that of controls in pregnancies destined for early pre‐eclampsia [1]. Normal PlGF results demonstrate high negative predictive values, suggesting that early second trimester PlGF testing can reassure clinicians and patients about the likelihood of adverse outcomes.

Although there was no statistically significant association between low PlGF and birthweight < 2500 g, the average birthweight was lower in the low PlGF group, likely reflecting the higher rate of preterm birth.

Strengths of this study include the use of gestational age specific centiles and a large cohort spanning four calendar years. Limitations include missing data for BMI, diabetes, and birthweight, as well as the absence of ethnicity information. Furthermore, the reference centiles used to classify PlGF levels were derived from an exclusively Caucasian population, which may limit applicability to diverse groups [5]. As a retrospective chart review, this study is also subject to inherent design limitations. Prospective multicentre studies with diverse participants are needed to validate cutoffs and evaluate the integration of PlGF testing into clinical care pathways.

Low PlGF (< 10th centile for gestational age) measured between 12 + 0 and 20 + 6 weeks is associated with increased risk of pre‐eclampsia and preterm birth in a high prevalent diabetic population. Early PlGF testing may help stratify risk and guide surveillance, especially in settings where early pregnancy screening is not universally available. Normal PlGF results have a high negative predictive value and could reassure clinicians and patients. Gestational age adjusted PlGF measurement, rather than fixed thresholds or the sFl‐t1/PlGF ratio, remains the preferred approach to early identification of placental dysfunction [3].

## Author Contributions

**Genevieve M. Dietrich:** data collection, analysis and interpretation of data, manuscript drafting and final approval of version to be sent. **Adrielle P. Souza Lira:** conception and design of the study, analysis and interpretation of data, manuscript drafting and final approval of version to be sent. **Eman Ramadan:** analysis and interpretation of data, manuscript drafting and final approval of version to be sent. **Adewumi Adanlawo:** analysis and interpretation of data, manuscript drafting and final approval of version to be sent. **Ernesto A. Figueiro‐Filho:** main PI, conception and design of the study, analysis and interpretation of data, manuscript drafting and final approval of version to be sent.

## Conflicts of Interest

The authors declare no conflicts of interest.

## Data Availability

The data that support the findings of this study are available on request from the corresponding author. The data are not publicly available due to privacy or ethical restrictions.
